# Follow up of natural infection with *Trypanosoma cruzi* in two mammals species, *Nasua narica* and *Procyon lotor* (Carnivora: Procyonidae): evidence of infection control?

**DOI:** 10.1186/1756-3305-7-405

**Published:** 2014-08-29

**Authors:** Fernando Martínez-Hernández, Emilio Rendon-Franco, Lilia María Gama-Campillo, Claudia Villanueva-García, Mirza Romero-Valdovinos, Pablo Maravilla, Ricardo Alejandre-Aguilar, Nancy Rivas, Alex Córdoba-Aguilar, Claudia Irais Muñoz-García, Guiehdani Villalobos

**Affiliations:** Departamento de Ecología de Agentes Patógenos, Hospital General Dr. Manuel Gea González, Mexico City, México; Departamento de Producción Agrícola y Animal, UAM-Unidad Xochimilco, Calzada del Hueso 1100, Col. Villa Quietud, Delegación Coyoacán, CP 04960 México; División Académica de Ciencias Biológicas, Laboratorio de Ecología del Paisaje y Cambio Global, Universidad Juárez Autónoma de Tabasco, Villahermosa, Tabasco, México; Departamento de Biología Molecular e Histocompatibilidad, Hospital General Dr. Manuel Gea González, Mexico City, México; Departamento de Parasitología, Laboratorio de Entomología, Escuela Nacional de Ciencias Biológicas, Instituto Politécnico Nacional, Mexico City, Mexico; Departamento de Ecología Evolutiva, Instituto de Ecología, Universidad Nacional Autónoma de México, Ciudad Universitaria, Apdo. Postal 70-275, 04510 México D.F, México

**Keywords:** *Trypanosoma cruzi*, Reservoir, *Procyon lotor*, *Nasua narica*, Infection control, Mexico

## Abstract

**Background:**

A large variety of mammals act as natural reservoirs of *Trypanosoma cruzi* (the causal agent of Chagas disease) across the American continent. Related issues are infection and parasite burden in these reservoirs, and whether they are able to control *T. cruzi* infections. These parameters can indicate the real role of mammals as *T. cruzi* reservoirs and transmitters. Here, two species of mammals, white-nosed coati (*Nasua narica*) and raccoon (*Procyon lotor*), were examined for to determine: a) *T. cruzi* presence, and; b) their ability to control *T. cruzi* infection.

**Methods:**

Multiple capture-recaptures of both species were carried out in semi-wild conditions in Villahermosa, Tabasco, Mexico, for 5 years. Two samplings per year (summer and winter) took place. Prevalence and pattern of *T. cruzi* infection were determined by PCR from both mammals’ blood samples.

**Results:**

Raccoon samples had a higher relative infection values (26.6%) compared to those of white-nosed coati (9.05%), being this difference significant in summer 2012 (*P* < 0.00001), summer (*P* = 0.03) and winter 2013 (*P* = 0.02). Capture and recapture data indicated three infection dynamics: 1) negative–positive-negative infection; 2) positive–negative-positive infection; and 3) positive at all sampling times.

**Conclusions:**

These results indicate that both coati and raccoons may be able to control *T. cruzi* infection. Thus, the role as efficient reservoirs could be questioned (at least for those times when mammals are able to tolerate the infection). However, while infected, they may also be able to approach human dwellings and play a role important in linking sylvatic and domestic cycles.

## Background

Chagas disease, caused by *Trypanosoma cruzi,* is one of the most important parasitic infections in Latin America. This disease is a zoonosis that occurs not only in humans but also other mammals, with the latter acting as natural reservoir hosts [1]. The parasite is primarily transmitted to vertebrate host by the feces of blood-sucking triatomine bugs, but the infection in humans can also occur via blood transfusion, organ transplant, or vertical transmission from mother to offspring [2]. In sylvatic hosts, the oral route of transmission seems to be the first infection mechanism among wild mammals [3, 4].

A large variety of mammals has been reported as natural reservoir of *T. cruzi* across the American continent. In 1912, an armadillo was the first sylvatic reservoir host reported by Chagas; gradually, more sylvatic reservoir animals of *T. cruzi* were discovered providing evidence for an enzootic cycle of parasite [5, 6]. Wild reservoirs of epidemiological importance include some edentates, marsupials, and rodents that, according to their habits and favorable local conditions (deforestation, weeding), play a significant role during the sylvatic and domestic cycles of the parasite [2]. In Mexico, different wild reservoir species are naturally infected with *T. cruzi*, including opossum (*Didelphis marsupialis* and *D. virginiana*), rodents (*Mus musculus*, *Sigmodon hispidus* and *Baiomys musculus*), bats (*Artibeus jamaicensis*) and, xenarthras (*Dasypus nomencinctus*) [7–9].

One neglected topic in Chagas disease research is that of whether infected animals are capable of controlling the parasite. Experimental evidence in dogs, for example, has shown that animals can control *T. cruzi* infections [10, 11]. Although the physiological mechanisms of how animals can exert such control are not clear, this line of research has not been explored. One issue where filling in this information can be useful is to understand the roles of reservoirs in the dynamics of infection in the whole transmission cycle. For example, if wild reservoirs are capable of controlling the parasite, then they can be parasite free for some time until the next re-infection takes place. However, preliminary studies of whether re-infection takes place in the wild need to be done.

Due to constant human invasion to sylvatic areas, a number of mammals are considered important synantropic reservoirs of *T. cruzi*. One case is that of opossums as these are extremely adaptable animals [7, 12]. Nevertheless, the role of others mammals in sylvatic or peridomestic cycles of transmission of *T. cruzi* is not well known. For example, in the occidental part of Mexico, wild triatomine populations were highly parasitized with *T. cruzi* and the principal feeding resource were *Dasypus nomencinctus* and *Procyon lotor*, suggesting that these mammals are key reservoirs in that region [13]. On the other hand, in Brazil and USA, different species of the Procyonidae family have also been reported to be infected with *T. cruzi* [14–17]. The Procyonidae family is widely distributed in the New World, from southern Canada to northern Argentina and in a wide variety of habitats that include desert, northern forest, tropical rainforest and wetland. Two species of this family are extensively distributed in Mexico: raccoon (*Procyon lotor*) and white-nosed coati (*Nasua narica*). Both species are common carnivores in terms of density and biomass in the neotropical forest [18, 19]. Using *P. lotor* and *N. narica* as study subjects the aims of our study were: a) to determine the infection by *T. cruzi*; and, b) to evaluate the ability to control *T. cruzi* infections. Our study was carried out for over a period of 5 years in semi-wild conditions.

## Methods

### Study area and animal population

The study was carried out at the Zoological Park “Parque Museo de La Venta” located in Villahermosa City, Tabasco State, Mexico (18°00′05.39′ N, 92°56′02.52′O, 17 masl). The area is damp tropical with a temperature range from 17.3 to 42.5°C, 80% relative humidity and annual rainfall from 2000 to 4000 mm^3^ (http://www.smn.cna.gob.mx). The park has an area of approximately 2.5 h and a disturbed forest relict of 4.3 h, surrounded by urban area. Free populations of raccoon (*Procyon lotor*) and white-nosed coati (*Nasua narica*) live inside the park. The abundance calculated by our working group was 98 ± 26.3 (mean ± SE) for raccoons and 108 ± 7.7 (mean ± SE) for coatis, using capture-recapture methodology (algoritm Lincoin-Petersen) with R-capture^®^ ver. 3.0.1 software. Animals feed from the local natural resources and also by what tourists provide. Additionally, coatis receive fruit, oat, bread and hard-boiled eggs from the park maintenance staff.

### Animal capture

Multiple captures were done from 2009 to 2013, with two samplings per year (summer and winter). Thus, 10 sampling episodes were carried out. Each sampling lasted ten days. Ten box traps (No. 108, Tomahawk Live Trap Company, Tomahawk, Wisconsin, USA) baited with canned sardine were used to capture raccoons. White-nosed coatis were captured with an anesthetic dart shot from 2 to 4 m of distance with a blowgun. Chemical restraint was done with 0.4 to 1.0 mL of 10% ketamine (Pisa-Agropecuaria, Guadalajara, Mexico) and 0.1 to 0.2 mL of 2% xylazine (Pisa-Agropecuaria, Guadalajara, Mexico) according to animal species and size. A blood sample was taken from jugular vein, while sex and age (categorized as adult or young based on size, weight and sexual characters) were recorded. Since 2010, each animal was tattooed with a consecutive number. 2009 samples were not considered for the follow up of infection but for parasite presence only. Approximately, 0.5 mL of blood sample was added to lysis buffer (50 mM of Tris pH 8, 50 mM of EDTA pH 8, 50 mM of NaCl, 20 μg/mL of RNAsa, 1% of SDS and 200 μg/mL of proteinase K) by DNA extraction [20]. Animals were released at the site of capture after total recovery from anesthesia.

### DNA isolation and PCR for *T. cruzi*presence

Determination of *T. cruzi* infection was performed by PCR [21]. Briefly, DNA extraction was performed according to the phenol/chloroform technique [22]. DNA concentration between 0.5 and 1 μg was used for each PCR volume of 25 μL. The master mix contained 2 U of *Taq polymerase*, 12.5 μM of dNTPs, 2 mM of MgCl_2_ and 0.1 mM of each primer. Miniexon primers were used whilst initial DNA denaturalization was carried out at 94°C for 5 min, followed by 35 cycles of DNA denaturalization (94°C), oligonucleotide alignment (68°C), and chain elongation (72°C), ending with a final elongation period at 72°C for 7 min. PCR products were separated and visualized on 1.5% agarose gels stained with ethidium bromide and observed under UV light. A *T. cruzi* sample isolated from a human collected in Oaxaca (Mexico) was used as positive control in all amplification reactions.

### Data analysis

Prevalence and infection differences between sex, age, species, and capture period were analyzed using Chi-Square test or Fisher’s test, with the level of significance set at *P* < 0.05, using Epidat 3.1^®^ software (Servicio de Epidemiología Dirección Xeral de Innovación e Xestión de Saúde Pública, Santiago de Compostela, Coruña, Spain). This software has been widely implemented to analyze prevalence and infection data [23, 24].

### Ethics statement

Animal manipulation was conducted following regulations by the Bioethics Committee of the Universidad Autónoma Metropolitana (DCBS.CICUAL.008.13) as well as legal permission of the Secretaría del Medio Ambiente y Recursos Naturales (Registration No. FAUT-0250).

## Results

### Relative infection per species

282 white-nosed coatis and 126 raccoons were captured and re-captured between summer 2009 to winter 2013. The same number of blood samples was analyzed by PCR. Raccoon samples showed a higher infection percentage than those of white-nosed coati samples, with significant differences for summer 2012 (*P <* 0.0001) and summer and winter 2013 (*P =* 0.03 and *P =* 0.02 respectively). Infection percentage was independent of whether an animal was captured or recaptured (white-nosed coati = 6% against to 11.81%; raccoons = 19.05% against to 36.95% between capture and recapture; both comparisons, *P >* 0.05).

### Prevalence of infection in both species

A high prevalence was observed for both species; nevertheless the raccoon samples showed a higher prevalence than those of coatis. Changes in *T. cruzi* prevalence along the study were observed from 0% to 33% for white-nosed coatis, and from 0 to 93% for raccoons. The highest prevalence were detected in summer 2012 and winter 2013 and these were significantly different from other periods; for coatis, summer 2012 was different to the others periods (*P ≤* 0.02) except for winter 2013 (*P >* 0.05), likewise this period (winter 2013) was different to summer 2009, 2010, 2013 and winter 2009, 2010 (*P ≤* 0.04); for raccoons, summer 2012 was different to all periods (*P <* 0.0001) and winter was different to the other periods (*P ≤* 0.01) except for summer 2013 (*P >* 0.05). Because of this difference by capture period, all the others comparisons (species, sex and age) were done by specific capture period.

### Follow up of infection by T. cruzi over a period of 5 years

In white-nosed coati, the infection with *T. cruzi* was detected in summer of 2010 while in raccoons, infection was determined one and half years later (winter 2011). Interestingly, infection was not detected in both species in 2009. For white-nosed coatis, the predominant period of infection was summer, although for the 2013 this observation was the opposite (Table 1). For raccoons, since the infection was detected, individuals were always infected which was especially the case for re-captures. Again for raccoons, in only two periods (summer 2012 and winter 2013), first time captures were positive (Table 1).

**Table 1:** ***Trypanosoma cruzi*** **infection in captures and recaptures of** ***Nasua narica*** **and** ***Procyon lotor*** **during five years**

	S, 2009*	W, 2009*	S, 2010	W, 2010	S, 2011	W, 2011	S, 2012	W, 2012	S, 2013	W, 2013	Total
***Nasua narica***											
***n***	31	41	34	30	24	15	30	33	23	21	210
**PCR +**	0	0	1	0	2	0	10	2	0	4	9.05 (19/210)
**% PCR +**, ^®^			0	0 (0/13)	7.69 (1/13)	0 (0/12)	37.50 (6/16)	6.67 (2/30)	0 (0/11)	26.67 (4/15)	11.81 (13/110)
**% PCR +, ©**			2.94 (1/34)	0 (0/17)	9.09 (1/11)	0 (0/3)	28.57 (4/14)	0 (0/3)	0 (0/12)	0 (0/6)	6 (6/100)
***Procyon lotor***											
***n***	10	7	7	10	14	16	15	17	11	19	109
**PCR +**	0	0	0	0	0	1	14	2	2	10	26.60 (29/109)
**% PCR +**, ^®^			0	0 (0/3)	0 (0/3)	20 (1/5)	88.89 (8/9)	25 (2/8)	22.20 (2/9)	34.44 (4/9)	36.95 (17/46)
**% PCR +, ©**			0 (0/7)	0 (0/7)	0 (0/11)	0 (0/11)	100 (6/6)	0 (0/9)	0 (0/2)	60 (6/10)	19.05 (12/63)

^®^,Re-captures; ©, Captures; S, Summer; W, Winter.

*During the first two periods, animals were not tattooed. These samples were only considered for detecting *T. cruzi*.

Positivity to infection for animals captured and re-captured in different periods of time is described in Table 2. Three white-nosed coatis and eleven raccoons *T. cruzi* positive were captured only once (e.g. C3 and, C13; M41 and, M46, respectively). Although some animals were captured and re-captured in different periods before the infection was detected for the first time, the follow up of infection was not carried out later because these animals were not re-captured again (eight individuals for coatis and raccoons, e.g. C8 and, M32 respectively). In those animals whose follow up infection was done, three dynamics of infection were observed: 1) negative–positive-negative infection (three coatis C56, C83 and C84; and four for raccoons M19, M37, M40 and, M42); 2) positive–negative-positive infection: (one coati C79; one raccoon, M15); and 3) individuals that remained infected (one coati, C69; and one raccoon, M53). There was no significant difference between the sexes for both species (*P > 0.05*).

**Table 2:** ***Trypanosoma cruzi*** **infection and follow up of infection in** ***Nasua narica*** **and** ***Procyon lotor*** **during eight periods of capture**

	Host	Code _Sex_Age	Periods of capture							
	***Nasua narica***		S, 2010	W, 2010	S, 2011	W, 2011	S, 2012	W, 2012	S, 2013	W, 2013
1		C3_F_A			✚ ©					
2		C8_F_A	─ ©			─ ^®^		─ ^®^		✚ ^®^
3		C13_M_Y	✚ ©							
4		C17_M_A	─ ©	─ ^®^	✚ ^®^					
5		C21_F_A	─ ©	─ ^®^	─ ^®^		✚ ^®^			
6		C29_M_A	─ ©				✚ ^®^			
7		C35_M_A	─ ©				✚ ^®^			
8		C37_F_A	ND ©		─ ^®^	─ ^®^	✚ ^®^			
9		C56_M_A		─ ©			✚ ^®^	─ ^®^		
10		C63_M_A			─ ©			─ ©		✚ ^®^
11		C69_M_A				─ ©	✚ ^®^	✚ ^®^		✚ ^®^
12		C74_F_A					─ ©	✚ ^®^		
13		C79_F_A					✚ ©	─ ^®^		✚ ^®^
14		C81_F_A					✚ ©			
15		C83_M_Y					✚ ©	─ ^®^		
16		C84_M_A					✚ ©	─ ^®^		
	*Procyon lotor*									
1		M4_F_A		─ ©			✚ ^®^			
2		M11_M_A		─ ©			✚ ^®^			
3		M15_M_A		─ ©	─ ^®^	─ ^®^	✚ ^®^		─ ^®^	✚ ^®^
4		M16_F_A			─ ©				✚ ^®^	
5		M18_M_A			ND ©	─ ^®^	✚ ^®^			
6		M19_F_A			─ ©	─ ^®^	✚ ^®^	─ ^®^	─ ^®^	
7		M32_F_A				─ ©		─ ©	─ ^®^	✚ ^®^
8		M33_M_A				─ ©	✚ ^®^			
9		M34_M_A				─ ©	✚ ^®^			
10		M37_F_A				─ ©		✚ ^®^	─ ^®^	
11		M40_M_A				─ ©	✚ ^®^	─ ^®^	─ ^®^	─ ^®^
12		M41_F_A					✚ ©			
13		M42_M_A					✚ ©	✚ ^®^	─ ^®^	
14		M43_F_A					✚ ©			
15		M44_M_A					✚ ©			
16		M45_F_A					✚ ©			
17		M46_M_A					✚ ©			
18		M53_M_A						─ ©	✚ ^®^	✚ ^®^
19		M56_F_A								✚ ©
20		M58B_F_A								✚ ©
21		M5b_F_Y								✚ ©
22		M59_F_A								✚ ©
23		M61_M_Y								✚ ©
24		M64_F_Y								✚ ©
25		M90_F_A							─ ©	✚ ^®^

^®^, Re-captures; ©, captures; S, Summer; W, Winter; A, Adult; Y, Young; F, Female; M, Male; +, Positive PCR; -, Negative PCR; ND, No determined.

## Discussion

In Mexico, as well as in other countries, few studies on reservoirs of *T. cruzi* have been carried out [7–9]. Thus, our study provides key information on the role of reservoirs (*Nasua narica* and *Procyon lotor*) in the transmission and maintenance of *T. cruzi* in Mexico. This is complemented with the follow up of infection which took place for a relatively long period (five years). In this sense, the dynamic of *T. cruzi* infection is unknown. This is, it is unclear whether some species maintain longer and/or higher parasitemias than others. In relation to this, we found that the *T. cruzi* infection percentage was higher in raccoons (26.6%) than in white-nosed coatis (9%). Such difference was significant in three periods of captures when high prevalence was detected. Raccoons (*P. lotor*) are described as important reservoirs in the USA. Extensive seroprevalence surveys showed infection rates that range from 3 to 68% [25, 26]. A similar situation was found in Brazil as there was a high *T. cruzi* parasite burden in crab-eating raccoons (*P. cancrivorus*; 15%) and coatis (*Nasua nasua*; 29%) which contrasted with other mammals such as crab-eating foxes (*Cerdocyon thous*) and ocelots (*Leopardus pardalis*) (0% parasite burden for both species) [17]. Due to its biomass, high parasite burden and distribution, *N. nasua* seems to play a role in the maintenance and dispersion of *T. cruzi*, acting as a “bioaccumulator” for different *T. cruzi* genotypes (Discrete Typing Units) as has been discussed for Brazil [4, 17].

Unlike the above studies of mammals from Brazil, we found that *P. lotor* presented a high *T. cruzi* infection, which was somehow similar to infection percentages observed for the same species in the USA. The differences between the infection percentages in *N. narica* and *P. lotor* could be related to their susceptibility to *T. cruzi* infection. In a study carried out in two didelphid species (naturally and experimentally infected with *T. cruzi*), serological titers of naturally infected *P. opossum* showed significant individual variation, while those of *D. marsupialis* remained stable during the entire follow-up period [12]. On the other hand, the serological titers of the experimentally infected animals varied according to the inoculated strain [12]. Although both species were able to mount efficient humoral immune responses, the inter-specific differences probably reflected distinct strategies selected by these two species during their coevolution with *T. cruzi* [12]. Whether this is also the case for our study species, it remains to be investigated.

Using PCR, new infections with *T. cruzi* were detected in both species. This was observed in the majority of the captures and re-captures, indicating a dynamic transmission of *T. cruzi* in these populations. The follow up of infection suggest that animals could be able to control the infection and re-infections. That animals may be able to control *T. cruzi* infection has been already documented but using laboratory studies. One case is that of dogs infected with *T. cruzi* that were able to control the parasite within 50 days post-infection [10]. Furthermore, when dogs were re-infected they showed again parasitemia, yet this was lower than that of the first infection. In another study also with dogs, no parasitemia was observed when animals were re-infected [11]. Using a related parasite, infection with *Trypanosoma evansi* in *N. nasua* showed a high parasitemia in the first month post-infection, with a further decrease until the parasite was no longer detected [27]. Instead, no correlation was detected with humoral response in these studies, because antibodies were detected at high titers until the end (255 day for *N. nasua*; 38 months for dogs), [11, 27], suggesting that in wild captured animals, they could be considered positive only by serological tests but without parasites presence. Thus, this evidence pieces provide support to the hypothesis of control of infection and re-infections in *N. narica* and *P. lotor.* Moreover, during the last three periods only recaptures were positive, indicating that no new individuals had been infected. Dynamic of the infection i.e. infection-reinfection or continuum infection has strong implications for the reservoir status of the different species, because the role as efficient reservoir could be questioned. However, while infected, they may also be able to approach human dwellings and play a role important in linking sylvatic and domestic cycles.

How do our study animals become infected? This can be related to the possibility that both mammals feed directly on infected triatomines and thus become infected. This behavior has been already demonstrated in the carnivores striped skunk (*Mephitis mephitis*) and raccoon (*P. lotor*) [28, 29]. Observational data indirectly indicate that triatomines can be an important source of food for our study subjects. In a survey using feces from *N. narica*, it was shown that coatis consumed predominantly fruit (46.05%), arthropods (39.07%) and vertebrates (6.98% mammals, 6.51% reptiles, 1.39% birds) [19]. Moreover, arthropods became a key source of food consumed during the wet season (July-October) with vertebrates being less consumed during this season, in contrast with the dry season (November-June) [19]. Interestingly, we observed that from 2010 to 2012, all coati individuals positive to *T. cruzi* were those from summer, a season where an increased consumption of invertebrates was also noted (all authors’ unpublished data). Additionally, although, the search of triatomines was not exhaustive, some adults of *Triatoma dimidiata* infected with *T. cruzi* were collected in the area. Further studies should focus on investigating whether triatomines are not only consumed but are also the source of *T. cruzi* infection for *N. narica* and *P. lotor.*

## Conclusions

Studies on wild animals as real or potential reservoir of *Trypanosoma cruzi* are scarce. In this sense, the capacity of these animals to maintain and transmit the parasite is unclear. Previous experimental evidence, and our findings, indicate that coatis and raccoons are able to control *T. crzui* infection. If this is indeed the case, their role as efficient reservoirs could be questioned. These findings are particularly important because these species may also be able to approach human dwellings and play a role important in linking sylvatic and domestic cycles when they are infected. However, more studies must be conducted to determine the relationship of these species or risk to human population surrounding.
